# Effects of a multispecies synbiotic on glucose metabolism, lipid marker, gut microbiome composition, gut permeability, and quality of life in diabesity: a randomized, double-blind, placebo-controlled pilot study

**DOI:** 10.1007/s00394-019-02135-w

**Published:** 2019-11-15

**Authors:** Angela Horvath, Bettina Leber, Nicole Feldbacher, Norbert Tripolt, Florian Rainer, Andreas Blesl, Markus Trieb, Gunther Marsche, Harald Sourij, Vanessa Stadlbauer

**Affiliations:** 1 Division of Gastroenterology and Hepatology, Medical University of Graz, Auenbruggerplatz 15, 8036 Graz, Austria; 2 Center for Biomarker Research in Medicine (CBmed), Stiftingtalstrasse 5, 8010 Graz, Austria; 3 Division of Transplantation Surgery, Medical University of Graz, Auenbruggerplatz 29, 8036 Graz, Austria; 4 Division of Endocrinology and Diabetology, Medical University of Graz, Auenbruggerplatz 15, 8036 Graz, Austria; 5 Division of Pharmacology, Otto Loewi Research Center, Medical University of Graz, Universitätsplatz 4, 8010 Graz, Austria; 6 Zayed Center for Health Sciences (ZCHS), UAE University, Al-Ain, UAE

**Keywords:** Diabetes mellitus, Type 2, Glycated haemoglobin, Zonulin, Lipopolysaccharides, Short-Form 36

## Abstract

**Purpose:**

Diabesity, the combination of obesity and type 2 diabetes, is an ever-growing global health burden. Diabesity-associated dysbiosis of the intestinal microbiome has gained attention as a potential driver of disease and, therefore, a possible therapeutic target by means of pro- or prebiotic supplementation. This study tested the effects of a multispecies synbiotic (i.e. a combination of probiotics and prebiotics) on glucose metabolism, gut microbiota, gut permeability, neutrophil function and quality of life in treatment-experienced diabesity patients.

**Methods:**

A randomized, double-blind, placebo-controlled pilot study with 26 diabesity patients was conducted in which patients received a daily dose of a multispecies probiotic and a prebiotic (or a placebo) for 6 months.

**Results:**

There were no changes in glucose metabolism or mixed meal tolerance test responses throughout the study. The analysis of secondary outcomes revealed beneficial effects on hip circumference [− 1 (95% CI − 4; 3) vs +3 (− 1; 8) cm, synbiotics vs. placebo, respectively, *p *= 0.04], serum zonulin [− 0.04 (− 0.2; 0.1) vs +0.3 (− 0.05; 0.6) ng/ml, *p *= 0.004)] and the physical role item of the SF36 quality of life assessment [+ 5.4 (− 1.7; 12.5) vs − 5.0 (− 10.1; 0.2) points, *p *= 0.02] after 3 months of intervention, and lipoprotein (a) [− 2.1 (− 5.7; 1.6) vs +3.4 (− 0.9; 7.9) mg/dl, *p *= 0.02] after 6 months. There were no significant differences in alpha or beta diversity of the microbiome between groups or time points.

**Conclusions:**

Glucose metabolism as the primary outcome was unchanged during the intervention with a multispecies synbiotic in patients with diabesity. Nevertheless, synbiotics improved some symptoms and biomarkers of type 2 diabetes and aspects of quality of life suggesting a potential role as adjuvant tool in the management of diabesity.

**Graphic abstract:**

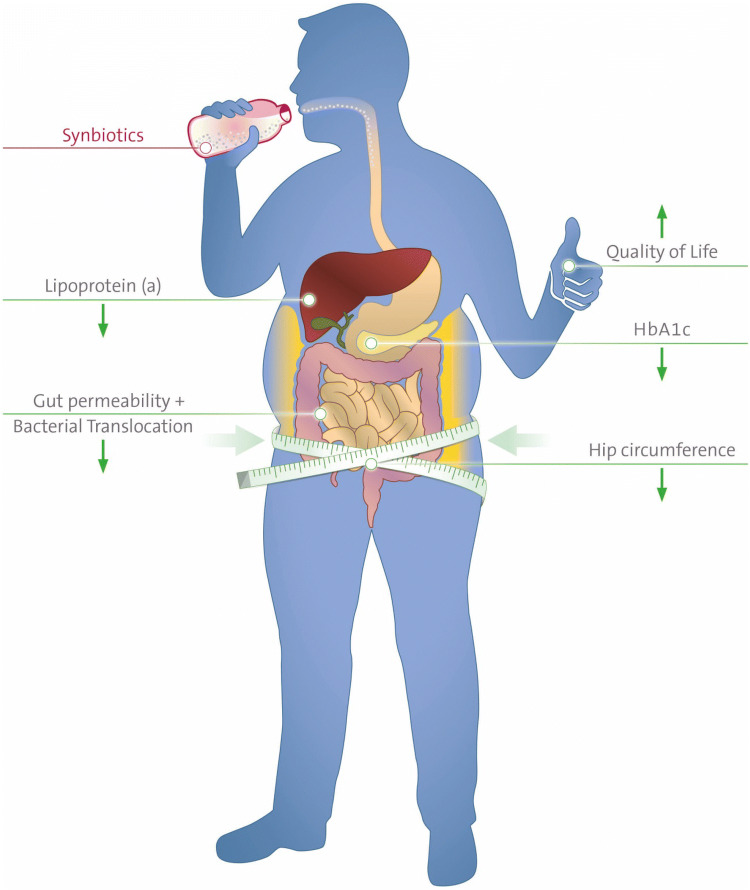

**Electronic supplementary material:**

The online version of this article (10.1007/s00394-019-02135-w) contains supplementary material, which is available to authorized users.

## Introduction

Diabetes mellitus type 2 (T2D) is a long-term metabolic disorder characterized by high blood glucose levels, beta-cell dysfunction and insulin resistance, with a steeply increasing global prevalence [1]. The global obesity epidemic is strongly associated with the high prevalence of T2D, coining the term “diabesity” [2]. T2D is a complicated and costly disease to treat and health care costs are directly related to the patients’ degree of obesity and associated complications [3]. The constant need for care and the fear of debilitating complications take a toll on patients’ quality of life [4].

In search for new potential therapies, the microbiota-gut-pancreatic axis gained attention [5]. Patients with T2D show a marked reduction of short-chain fatty acid (SCFA) producing bacteria [6, 7]. SCFA can act anorectic, lead to the secretion of glucagon-like peptide-1 and, therefore, directly influence insulin sensitivity and glycaemia [8, 9]. Furthermore, SCFA is a nutrient source for enterocytes and promotes gut barrier function [10–13]. Accordingly, patients with T2D show increased gut permeability and signs of intestinal injury [14–17]. Lipopolysaccharide (LPS) can translocate through the damaged gut barrier, act as a potent inflammatory mediator, influence insulin sensitivity and disturb the functionality of the innate immune system [18–20].

Because of the proposed association between T2D and the microbiome, probiotic modulation has been considered as a possible therapeutic approach in T2D. Meta-analyses showed that probiotics have been successfully used to improve classical traits of T2D, such as fasting plasma glucose, insulin concentration, insulin resistance, and glycated haemoglobin (HbA1c) [21–23]. Furthermore, lipid metabolism markers, such as high-density lipoprotein-cholesterol (HDL-C), total cholesterol and triglycerides, have been positively influenced during probiotic supplementation; however, reports are not conclusive [24, 25]. Especially interventions over an 8-week period, with probiotics of the genus *Lactobacillus* and/or multispecies formulations procured promising results [26, 27]. Prebiotics (i.e. indigestible dietary compounds that increase the growth and activity of fibre fermenting bacteria) are linked to the increase of SCFA-producers in the intestine and have also been shown to improve glucose metabolism and meal handling in T2D [28–31]. Combinations of pro- and prebiotics, known as synbiotics, can exert beneficial effects on glycemic control and oxidative stress [32–35]. Thus, prebiotics might complement the effects of probiotics in diabesity patients.

A recent trial in obese, post-menopausal, non-diabetic women showed that a combination of probiotic strains (EcologicBarrier^®^, Winclove, The Netherlands) could improve markers of insulin resistance, lipid profile and anthropometric measurements in a dose dependent manner [36]. The same product was tested in treatment-naïve T2D patients and showed improved insulin sensitivity after 6 months of intervention [37]. While this probiotic formulation showed beneficial effects in obese subjects and treatment-naïve diabetic patients, its potential has not been assessed in patients in more progressed T2D. Diabetes therapy and complications of the disease might alter the potential effect of the intervention. We tested the effects of a 6-month intervention with this commercially available multispecies probiotic (EcologicBarrier^®^, Omnibiotic Hetox^®^) in combination with a prebiotic (OmniLogic^®^ Plus, Institute Allergosan, Graz, Austria) to potentially improve the efficacy of the probiotic alone on glucose metabolism in a randomized, double-blind, placebo-controlled pilot trial in treatment-experienced, obese T2D patients. In addition, we focused on changes of gut microbiota, gut permeability, lipid markers, neutrophil function and quality of life during the intervention.

## Methods

### Trial design

Between October 2015 and March 2017, all outpatients of the Division of Endocrinology and Diabetology at the University Hospital Center Graz, Austria, who showed signs of diabesity, were informed about the study. If they were generally interested to participate, they were screened for eligibility. All patients gave written informed consent prior to screening, were older than 18 years and committed to long-term follow-up. The study was approved by the Research Ethics Board of the Medical University of Graz (26-464 ex 13/14) and was registered prior to the inclusion of the first patient at clinicaltrials.gov (NCT02469558). All study procedures were performed according to the Declaration of Helsinki and Good Clinical Practice. All patients had diagnosed T2D, a body mass index (BMI) of 30–40 kg/m^2^, HbA1c above 6.5% (48 mmol/mol), stable diabetes therapy for at least 6 months and fulfilled none of the following exclusion criteria: Type 1 diabetes, Maturity Onset Diabetes of the Young, secondary diabetes due to a specific disease or glucocorticoid therapy, pregnancy, hypothalamic cause of obesity, Cushing syndrome, major psychiatric diseases including diagnosed eating disorders, history of drug and alcohol abuse, history of bariatric surgery, use of probiotics at baseline, antibiotic therapy within the last 4 weeks of inclusion, inflammatory bowel disease, pancreatitis, chronic non-steroidal anti-inflammatory drug treatment, glucagon-like-peptide (GLP)-1 receptor agonist therapy or acarbose therapy, recent (< 12 weeks) acute myocardial infarction or decompensated heart failure, recent stroke, known malignancy or any other condition or circumstance, which (in the opinion of the investigator) would affect the patients ability to adhere to the study protocol.

Patients were stratified for the use of dipeptidyl peptidase (DPP)-4 inhibitors and randomly allocated into two groups in a ratio of 1:1. Randomization with permutated blocks was done by the study coordinators in accordance with the principal investigator using the online software tool Randomizer^®^ (Institute of Medical Informatics, Medical University of Graz, Austria). The synbiotics group received a daily dose of a multispecies probiotic and a prebiotic for 6 months, and the control group received an equal amount of similar looking and tasting placebos. The probiotic used in this study was Ecologic Barrier^®^ (Winclove, Amsterdam, The Netherlands) which is marketed as Omnibiotic Hetox^®^ (Institut Allergosan, Graz, Austria) in Austria, Germany and Switzerland. Each dose contains a total of approximately 1.5 × 10^10 CFU of a blend containing *B. bifidum W23*, *B. lactis W51*, *B. lactis W52, L. acidophilus W37*, *L. casei W56*, *L. brevis W63*, *L. salivarius W24*, *Lc. lactis W58* and *Lc. lactis W19* in 6 g of matrix (maize starch, maltodextrins, vegetable protein, potassium chloride, magnesium sulphate, amylases and manganese sulphate). The matrix without bacteria was used as placebo. The probiotic/placebo powder was dispensed in sachets which the patients dissolved every morning in 250 ml of water and drank after 10 min of activation time. The prebiotic was Omnilogic Plus (Institut Allergosan, Graz, Austria), containing Galacto-oligosaccharides P11 (GOS) and Fructo-oligosaccharides P6 (FOS), konjac glucomannan P13 (E425), calcium carbonate (E170), zinc citrate 3-hydrate, vitamin D3 (cholecalciferol) and vitamin B2 (riboflavin) (E101) and a matrix containing maltodextrin, natural elderflower flavouring and Gum Arabic (E414). A daily dose of 10 g (equivalent to 8 g of active prebiotics) was dissolved in 250–500 ml of water and taken in the evening. The matrix was used as a placebo. Both types of placebo were produced by Winclove (Amsterdam, The Netherlands). Patients were instructed not to change their dietary habits and physical activity habits during the study period. Dietary habits were monitored using an extensive, validated food frequency questionnaire [38].

Patients, caregivers, investigators and outcome assessors were blinded to the allocation. An allocation list was kept by an independent trial pharmacist and disclosed after all the endpoints were assessed. To ensure blinding, the study products were packaged in consecutively numbered but otherwise blank sachets (probiotics) or containers (prebiotics).

Patients were included in the study for 1 year. In the first 6 months, patients were administered a daily dose of the synbiotics or placebo, in the second half-year patients were followed without study-specific intervention. During this year, four study visits were scheduled: at the beginning of the trial (baseline), after 3 months of intervention (3 months), at the end of treatment (6 months) and after the end of the follow-up period (12 months). Each visit, blood and stool samples were collected (more details are given in the online material) and clinical data were documented. In addition, patients underwent a mixed meal tolerance test (MTT) and answered quality of life related questionnaires at every study visit.

The primary endpoint of the study was glucose metabolism, assessed by HbA1c and area under the curve (AUC) of glucose and c-peptide during MTT. Secondary endpoints included the gut microbiome composition, gut permeability, lipid markers, neutrophil function and quality of life. This study was designed as a pilot trial to assess the effect of a synbiotic on the microbiota–gut–pancreatic axis. Sample size of 20 patients per group was chosen based on feasibility.

### Outcome assessments

#### Markers of metabolism

Glucose metabolism was characterized by HbA1c and the response to MTT. MTT was performed after an overnight fast. The patients were asked to ingest 10 kcal/kg body weight of a standard oral nutritional supplement (Fortimel compact, Nutricia, Erlangen, Germany), and blood was sampled simultaneously from a standard gauge cannula. Additional plasma samples were taken after 15, 30, 60 and 120 min. All samples were used to determine plasma glucose, insulin and c-peptide [39]. The following parameters were calculated: AUC for glucose and c-peptide, Matsuda index (IS_MTT_), quantitative insulin sensitivity check index (QUICKI), and first and second phase of insulin secretion, as previously described [40], as well as insulinogenic index (IGI) and early insulin response (EIR) [41].

All metabolic biomarkers were assessed by the certified routine biochemistry lab at the University Hospital Graz; details are given in the Online Resource. Additionally, as a functional lipid parameter, cholesterol efflux was measured using radioactivity assay as previously described [42].

#### Gut microbiome

Stool samples were collected on the day of the study visit or the evening before, kept at 4 °C until arrival at the hospital and then immediately frozen at − 80 °C. For microbiome analysis, DNA was isolated from the stool samples with the MagNA Pure LC DNA Isolation Kit III (Bacteria, Fungi) (Roche, Mannheim, Germany) according to manufacturer’s instructions. Hypervariable region V1–V2 of the 16S gene was amplified (primers: 27F-AGAGTTTGATCCTGGCTCAG; R357-CTGCTGCCTYCCGTA) and sequenced using Illumina Miseq technology (Illumina, Eindhoven, The Netherlands), as published before [43, 44].

Sequencing data were analysed using QIIME 2 tools on a local Galaxy instance (https://galaxy.medunigraz.at/) [45]. Denoising (primers removing, quality filtering, correcting errors in marginal sequences, removing chimeric sequences, removing singletons, joining paired-end reads, and dereplication) was done with DADA2 [46]. Taxonomy was assigned based on Silva 132 database release at 99% OTU level, trained using a Naïve Bayes classifier. To fit to the cutoff used for denoising in DADA2, sequencing-like reads were extracted from the Silva 132 database. Alpha diversity and richness were assessed with Shannon and Chao1 index, respectively. Beta diversity was examined by PCo-analysis based on Bray–Curtis dissimilarity and ANOSIM. Differences in composition between baseline and end of treatment were defined by Gradient Boosting Classifier, a machine learning algorithm, and ANCOM, implemented in QIIME2. Sequencing data are available at NCBI’s Sequencing Read Archive (SRA) under the BioProject ID PRJNA510713.

#### Gut permeability and bacterial translocation

Biomarkers of gut permeability (zonulin and diamine oxidase), LPS, bacterial DNA and LPS-related proteins [LPS-binding protein (LBP) and sCD14] were measured in serum. ELISA was used to measure zonulin, diamine oxidase (both: Immundiagnostik, Bensheim, Germany), LBP (Hycult, Uden, The Netherlands) and sCD14 (R&D Systems, Minneapolis, USA) according to the manufacturers’ instructions. Detection kits based on HEK-blue cells with TLR4 or TLR9 reporter cassettes (Invivogen, Toulouse, France) were used to assess LPS and bacterial DNA in serum, respectively. Protocols were adapted as previously described [47, 48].

#### Neutrophil function

Neutrophil function was assessed in heparinized whole blood. Phagoburst kit (Glycotope Biotechnology, Heidelberg, Germany) was used according to the manufacturer’s instructions to assess the production of reactive oxygen species by neutrophils (1) without stimulus (resting burst), (2) with fMLP as mild stimulus (priming) and with *E. coli* as strong stimulus (oxidative burst). Phagotest (Glycotope Biotechnology, Heidelberg, Germany) was used to assess phagocytic capacity and phagocytic activity of neutrophils. All tests are measured by flow cytometry evaluating 10,000 neutrophils.

#### Quality of life

Health-related quality of life (HR-QoL) was assessed by the Short Form (SF)-36 questionnaire and gastrointestinal quality of life index (GIQLI, Mapi Research Trust, Lyon, France) [49–51]. Both questionnaires were used in German and analysis was done according to the user manuals. Parameters of the SF-36 questionnaire were transformed to a scale of 0–100 on which higher numerical value equals better quality of life.

### Statistical analysis

Data are presented as count and percentage for categorical variables and as mean and 95% confidence interval for continuous variables. Categorical variables were compared between groups with Chi-square tests and continuous variables with Mann–Whitney *U* tests. To evaluate the effect of the intervention, differences from baseline were calculated and compared between groups with Mann–Whitney *U* tests. In addition to the comparisons between test group and control group, *L. brevis*^+^ patients (i.e. presence of *L. brevis* W63 in the microbiome after 6 months of intervention) were compared with *L. brevis−* patients (synbiotic and placebo treated). Associations between variables were evaluated using Spearman correlation. AUC was calculated with the trapezoidal rule (adjacent time intervals were evaluated separately and then summed up. Each interval was calculated by the mean of the values at the beginning and the end of the interval multiplied by the length of the interval in minutes). Analyses and visualization were performed using SPSS for Windows Version 23 (SPSS Inc., Chicago, IL, USA) and GraphPad Prism 6 (GraphPad Software, San Diago, USA). *p* values below 0.05 were considered significant.

## Results

### Patients’ characteristics

**Fig. 1:**
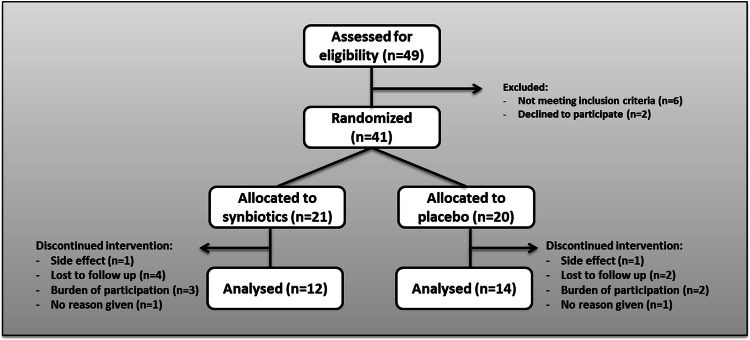
Consort flow diagram of enrolment

Of the 49 patients that were screened for eligibility, 41 were randomized, six did not meet the inclusion criteria and two declined to participate. Of the 41 randomized patients, 21 were allocated to the synbiotics group and 20 were allocated to the placebo group. All patients received the study product to which they were allocated. In the synbiotics group, 12 patients finished the study per protocol, one patient dropped out because of side effects (flatulence and diarrhoea), four patients were lost to follow-up, three patients were overstrained by the burden of participation, and one patient did not give a reason. In the placebo group, 14 patients finished the study per protocol, one patient dropped out because of side effects (flatulence and diarrhoea), two patients were lost to follow-up, two patients were overstrained by the burden of participation, and one patient did not give a reason. Enrolment details are summarized in Fig. 1. Of the seven serious adverse events documented during the study period, none was attributed to the intervention (Online Table 1). Groups were well balanced in terms of DPP4 inhibitor use, glucose metabolism and insulin resistance. Only one woman was allocated to the synbiotics group (8%), while six women (43%) were allocated to the placebo group; accordingly, weight, height and hip circumference was significantly lower in the placebo group, while HDL-C levels were significantly lower in the synbiotics group; BMI was comparable in both groups. Details are given in Table 1 and Online Table 2. Detailed information about glucose lowering medication is given in Online Table 3.

**Table 1:** Patient characteristics and changes during synbiotic intervention according to allocation; values are given as means (95% confidence interval)

	Synbiotics			Placebo			*P* values
	Baseline	3 months	6 months	Baseline	3 months	6 months	
*N*	12	–	–	14	–	–	–
Age (years)	61 (56; 65)	–	–	59 (54; 63)	–	–	*p *= 0.7^a^
Sex (female/male)	1/11 (8/92%)	–	–	6/8 (43/57%)	–	–	*p *= 0.08^a^
Use of DPP4 inhibitors	9 (75%)	–	–	8 (58%)	–	–	*p *= 0.3^a^
Height (cm)	179 (176; 183)	–	–	170 (164; 176)	–	–	*p *= **0.003**^**a**^
Weight (kg)	105 (99; 111)	101 (95; 108)	102 (98; 107)	98 (92; 104)	102 (96; 109)	101 (95; 106)	*p *= **0.03**^**a**^; *p *= 0.3^b^; *p *= 0.1^c^
BMI (kg/m^2^)	33 (31; 34)	34 (33; 36)	33 (32; 35)	34 (32; 36)	35 (32; 37)	35 (33; 37)	*p *= 0.6^a^; *p *= 0.3^b^; *p *= 0.5^c^
Waist circumference (cm)	119 (113; 125)	117 (112; 122)	117 (114; 121)	115 (111; 119)	116 (112; 120)	116 (112; 120)	*p *= 0.1^a^; *p *= 0.3^b^; *p *= 0.5^c^
Hip circumference (cm)	117 (112; 123)	116 (111; 122)	116 (113; 119)	111 (107; 115)	114 (109; 120)	114 (109; 120)	*p *= **0.04**^**a**^; *p *= **0.049**^**b**^; *p *= 0.2^c^
WHR	1.01 (0.98; 1.05)	1.01 (0.97; 1.05)	1.01 (0.99; 1.03)	1.04 (1.01; 1.07)	1.02 (0.98; 1.05)	1.02 (0.98; 1.07)	*p *= 0.5^a^; *p *= 0.7^b^; *p *= 0.8^c^

*DPP4 inhibitors* dipeptidyl peptidase-4 inhibitors, *BMI* body mass index, *WHR* waist to hip ratio

^a^Comparing baseline values between synbiotics and placebo group

^b^Comparing changes from baseline after 3 months of intervention between synbiotics and placebo group

^c^Comparing changes from baseline after 6 months of intervention between synbiotics and placebo group

### Microbiome analysis and *L. brevis*^+/−^

**Fig. 2:**
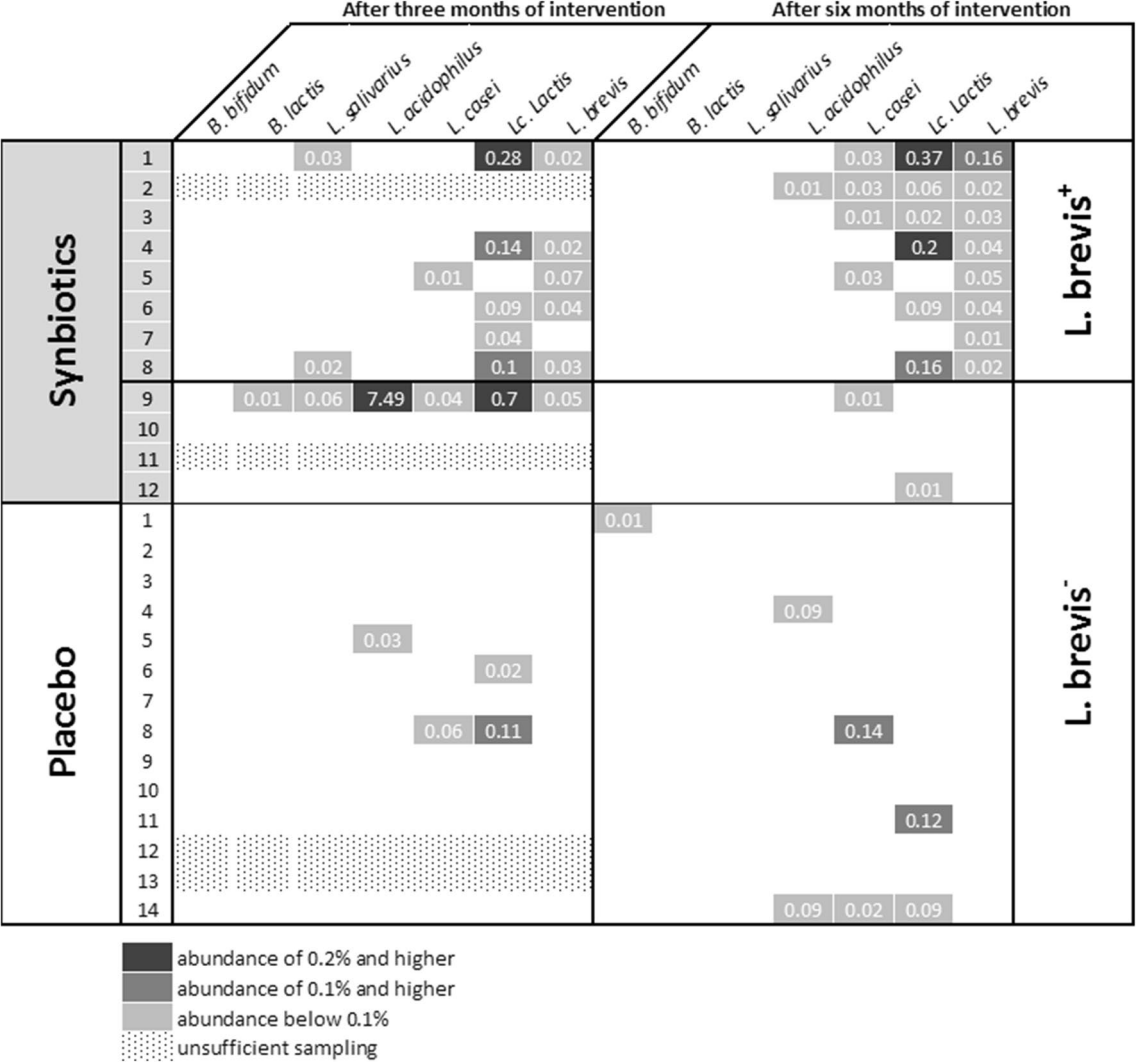
Abundance of bacteria included in the probiotic formulation after 3 and 6 months of intervention for individual patients. No statistically significant differences could be detected by ANCOM

Microbiome analysis did not show significant differences in taxa composition, alpha or beta diversity of the fecal microbiome between groups at any of the time points or between baseline and end-of-intervention in any group. Gradient boosting classifier identified the abundance of *L. brevis* as the most prominent difference between pre- and post-treatment microbiomes in the synbiotics group. This specific sequence was (a) part of the study product and (b) exclusively present during and at the end of intervention, not at baseline or follow-up. Therefore, its presence was likely due to the ingestion of the study product (*L. brevis W63*). At the end of the 6 months of intervention, 8 of 12 patients (67%) carried this sequence in their stool and were, therefore, defined as “*L. brevis*^+^” for a hypothesis generating, post hoc, subgroup analysis. Sequences corresponding to other strains of the product were recovered to a lesser degree. Details are given in Fig. 2.

### Glucose metabolism, lipid markers and anthropometrics

There was no significant change in glucose metabolism detected in the synbiotics group compared to the placebo group. Furthermore, response to MTT, insulin resistance and lipid profile did not change in either group throughout the study. Details are given in Table 2 and Online Table 2. Hip circumference was significantly reduced in the synbiotics group compared to the placebo group after 3 months of intervention [− 1 (95% CI − 4; 3) vs +3 (− 1; 8) cm, respectively, *p *= 0.04] (Table 1). Patients in the synbiotics group showed a reduction of lipoprotein (a) (LPA) after 6 months of intervention opposed to patients in the placebo group [− 2.1 (95% CI − 5.7; 1.6) vs +3.4 (− 0.9; 7.9) mg/dl, respectively, *p *= 0.01] (Online Table 2).

**Table 2:** Glucose metabolism markers and their changes during synbiotic intervention according to allocation; values are given as means (95% confidence interval)

	Synbiotics			Placebo			*p* values
	Baseline	3 months	6 months	Baseline	3 months	6 months	
HbA1c (mmol/mol)	64 (53; 74)	67 (54; 81)	67 (54; 80)	62 (59; 66)	69 (62; 69)	64 (58; 71)	*p *= 0.6^a^; *p *= 0.3^b^; *p *= 0.8^c^
FPG (mg/dl)	177 (147; 207)	188 (149; 228)	188 (142; 235)	174 (148; 200)	184 (162; 206)	163 (134; 191)	*p *= 0.9^a^; *p *= 0.4^b^; *p *= 0.5^c^
FPI (µU/ml)	23 (8; 38)	42 (3; 81)	62 (12; 111)	22 (13; 31)	25 (15; 34)	23 (13; 33)	*p *= 0.8^a^; *p *= 0.9^b^; *p *= 0.5^c^
C-peptide (ng/ml)	2.8 (1.8; 3.8)	2.4 (1.8; 3)	2.4 (1.7; 3.1)	1.7 (1.2; 2.3)	2 (1.5; 2.5)	10.1 (0; 28)	*p *= 0.05^a^; *p *= 0.1^b^; *p *= 0.3^c^
AUC_Glucose_ (g/dl) in minutes during MTT	28.1 (23.5; 32.7)	28.9 (22.7; 35.1)	27.8 (22.6; 33.1)	27.9 (25.2; 30.7)	28.6 (26.0; 31.2)	30.0 (23.9; 36.1)	*p *= 0.7^a^; *p *= 0.9^b^; *p *= 0.9^c^
AUC_insulin_ (µU/ml) in minutes during MTT	6690.4 (2644.2; 10,736.5)	4631.4 (1551.2; 7711.6)	10,776 (3810.8; 17,741.2)	5371 (3082.1; 7659.8)	6151.5 (3746.7; 8556.3)	5521.9 (2837.5; 8206.3)	*p *= 0.9^a^; *p *= 0.2^b^; *p *= 0.5^c^
AUC_c-peptide_ (ng/ml) in minutes during MTT	546.9 (414.8; 679.0)	471.1 (368.5; 574.4)	503.3 (389.5; 617.1)	445.7 (306.1; 585.4)	440.4 (318.4; 562.4)	469.7 (264.1; 675.3)	*p *= 0.3^a^; *p *= 0.2^b^; *p *= 0.9^c^

*HbA1c* glycated haemoglobin, *FPG* fasting plasma glucose, *FPI* fasting plasma insulin, *AUC* area under the curve, *MTT* mixed meal tolerance test

^a^Comparing baseline values between synbiotics and placebo group

^b^Comparing changes from baseline after 3 months of intervention between synbiotics and placebo group

^c^Comparing changes from baseline after 6 months of intervention between synbiotics and placebo group

**Fig. 3:**
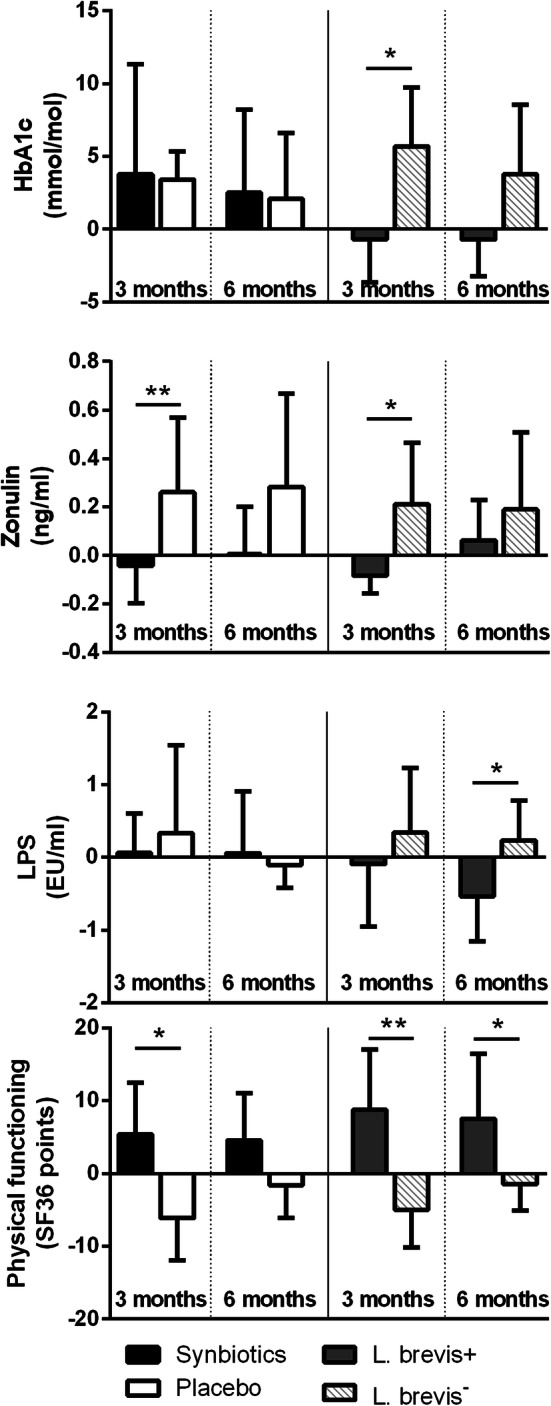
Significant changes in *L. brevis*^+^ patients. Parameters significantly changed in *L. brevis*^+^ patients are given for the synbiotics (*n *= 12) and placebo group (*n *= 14) as well as for *L. brevis*^+^ (*n *= 8) vs. *L. brevis*^−^ patients (*n *= 18). Values are given as mean changes to baseline with 95% confidence interval. HbA1c: glycated haemoglobin; LPS: lipopolysaccharide; *p < 0.05; **p < 0.01

In a post hoc analysis, *L. brevis*^+^ patients showed a decrease in HbA1c, while *L. brevis*^−^ patients showed an increase over the first 3 months [− 0.7 (95% CI − 3.6; 2.2) vs +3.4 (1.4; 5.4) mmol/mol, respectively, *p *= 0.03], similar patterns were observed after 6 months of intervention; however, the difference did not reach statistical significance. See also Fig. 3.

### Gut permeability and innate immune system

Serum zonulin showed a significant reduction after 3 months of intervention compared to the placebo group [− 0.04 (95% CI − 0.2; 0.1) vs +0.3 (− 0.05; 0.6) ng/ml, respectively, *p *= 0.004)]. *L. brevis*^+^ patients showed a significantly bigger reduction of serum zonulin levels compared to *L. brevis*^*−*^ patients [− 0.08 (95% CI − 0.16; − 0.01) vs +0.3 (− 0.05; 0.6) ng/ml, respectively, *p *= 0.03]. Serum zonulin at baseline correlated significantly with c-peptide (*r*_s_ = 0.424, *p *= 0.03), serum LPS levels (*r*_s_ = 0.522, *p *= 0.006) and bacterial DNA in serum (*r*_s_ = 0.425, *p *= 0.03). LPS levels were reduced in *L. brevis*^+^ patients versus *L. brevis*^−^ patients after 6 months of intervention [− 0.5 (95% CI − 1.2; 0.1) vs +0.2 (− 0.3; 0.8) EU/ml, respectively, *p *= 0.03]. Diamine oxidase, bacterial DNA in serum, sCD14 and LBP did not change significantly throughout the study. Neutrophils showed more resting burst after 3 months [2.5 (95% CI − 17.7; 22.7) vs − 20.2 (− 31.6; − 8.8) GMFI, respectively, *p *= 0.04] and more priming after 6 months of intervention in the synbiotics group compared to the placebo group [0.9 (95% CI − 0.8; 2.7) vs − 1.9 (− 3.5; − 0.3)%, respectively, *p *= 0.02]. Details are given in Table 3 and Fig. 3.

**Table 3:** Changes in gut permeability and bacterial translocation markers according to allocation; values are given as means (95% confidence interval)

	Synbiotics			Placebo			*p* value
	Baseline	3 months	6 months	Baseline	3 months	6 months	
Zonulin (ng/ml)	2.45 (2.04; 2.85)	2.40 (1.99; 2.81)	2.45 (2.04; 2.86)	2.01 (1.62; 2.41)	2.27 (1.98; 2.55)	2.29 (1.98; 2.61)	*p *= 0.2^**a**^; *p *= **0.004**^**b**^; *p *= 0.4^c^
Diamine oxidase (U/ml)	9.4 (6.2; 12.7)	8.9 (6.4; 11.5)	9.1 (5.7; 12.6)	8.8 (5.8; 11.8)	8.8 (5.0; 12.5)	9.4 (5.0; 13.7)	*p *= 0.8^a^; *p *= 0.8^b^; *p *= 0.7^c^
LPS (EU/ml)	0.64 (0.17; 1.10)	0.70 (0.19; 1.20)	0.69 (0; 1.69)	0.32 (0; 0.70)	0.73 (0; 1.92)	0.22 (0; 0.53)	*p *= 0.4^a^; *p *= 0.5^b^; *p *= 0.8^c^
Serum bacterial DNA (µmol/L)	4.38 (1.90; 6.85)	5.47 (0.49; 10.45)	3.21 (1.06; 5.36)	2.54 (0.46; 4.61)	6.38 (0; 15.3)	2.76 (0.49; 5.02)	*p *= 0.3^a^; *p *= 0.8^b^; *p *= 0.5^c^
LBP (ng/ml)	19.0 (17.5; 20.5)	17.1 (13.4; 20.8)	20.5 (17.5; 23.4)	19.5 (16.1; 22.9)	23.0 (15.8; 30.1)	21.6 (17.5; 25.7)	*p *= 0.9^a^; *p *= 0.1^b^; *p *= 0.7^c^
sCD14 (µg/ml)	1.6 (1.3; 1.9)	1.7 (1.5; 2.0)	1.5 (1.2; 1.9)	1.5 (1.2; 1.9)	1.4 (1.2; 1.6)	1.4 (1.2; 1.5)	*p *= 0.7^a^; *p *= 0.5^b^; *p *= 0.5^c^

*LPS* lipopolysaccharide, *LBP* LPS-binding protein

^a^Comparing baseline values between synbiotics and placebo group

^b^Comparing changes from baseline after 3 months of intervention between synbiotics and placebo group

^c^Comparing changes from baseline after 6 months of intervention between synbiotics and placebo group

### Quality of life

Patients in the synbiotics group showed significant improvement in physical functioning (SF-36) compared to the placebo group [+ 5.4 (95% CI − 1.7; 12.5) vs − 5.0 (− 10.1; 0.2) points, respectively, *p *= 0.02]. The difference was larger when comparing *L. brevis*^+^ to *L. brevis*^−^ patients and an improvement was observed over the entire intervention period [+ 8.8 (95% CI 0.5; 17.0) vs − 5.0 (− 10.1; 0.2) points, *p *= 0.003; and + 7.5 (95% CI − 1.4; 16.4) vs − 1.5 (− 5.1; 2.1) points, *p *= 0.03, respectively]. Other aspects of quality of life remained unchanged by the synbiotic intervention. Details are given in Table 4 and Fig. 3.

**Table 4:** Gastrointestinal quality of life (GIQLI) and health-related quality of life (HR-QoL) during synbiotic intervention according to allocation; higher values equal better quality of life; values are given as means (95% confidence interval)

GIQLI	Synbiotics			Placebo			*p* values
	Baseline	3 months	6 months	Baseline	3 months	6 months	
Emotions	16.5 (14.4; 18.6)	15.9 (13.3; 18.5)	16.9 (14.4; 19.4)	15.5 (13.6; 17.4)	16.3 (14.7; 17.8)	15.3 (13.0; 17.6)	*p *= 0.7^a^; *p *= 0.2^b^; *p *= 0.7^c^
Medical treatment	3.5 (2.8; 4.2)	3.4 (2.8; 4.0)	3.5 (2.9; 4.1)	3.8 (3.5; 4.1)	3.6 (3.1; 4.1)	3.7 (3.2; 4.2)	*p *= 0.5^a^; *p *= 0.9^b^; *p *= 0.9^c^
Physical function	19.6 (15.9; 23.3)	20.0 (16.2; 23.8)	19.9 (14.8; 25.0)	19.0 (15.1; 22.9)	18.8 (15.3; 22.3)	18.9 (15.6; 22.2)	*p *= 0.9^a^; *p *= 0.3^b^; *p *= 0.9^c^
Social function	13.9 (12.2; 15.6)	13.5 (11.4; 15.6)	13.9 (12.0; 15.8)	14.2 (12.7; 15.7)	14.1 (12.3; 15.9)	13.9 (12.3; 15.4)	*p *= 0.5^a^; *p *= 0.6^b^; *p *= 0.3^c^
Symptoms	64.3 (59.4; 69.2)	63.4 (58.4; 68.4)	61.8 (55.0; 68.6)	64.4 (59.9; 68.9)	64.7 (60.9; 68.5)	65.7 (61.5; 69.9)	*p *= 0.7^a^; *p *= 0.7^b^; *p *= 0.7^c^
Total score	117.8 (106.6; 129.0)	116.2 (103.6; 128.8)	116.0 (100.4; 131.6)	116.6 (106.7; 126.4)	117.4 (108.4; 126.5)	117.4 (107.9; 127.0)	*p *= 0.9^a^; *p *= 0.5^b^; *p *= 0.9^c^
HR-QoL							
General health	57.0 (45.9; 68.1)	58.2 (42.6; 73.8)	61.1 (45.7; 76.5)	61.1 (49.3; 72.9)	65.9 (56.5; 75.3)	67.1 (54.1; 80.1)	*p *= 0.2^a^; *p *= 0.8^b^; *p *= 0.7^c^
Social role	96.7 (89.1; 100)	90.0 (67.4; 100)	93.3 (83.3; 100)	86.7 (63.6; 100)	96.7 (89.1; 100)	90.0 (67.4; 100)	*p *= 0.8^a^; *p *= 0.8^b^; *p *= 0.6^c^
Physical role	80.0 (53.6; 100)	90.0 (67.4; 100)	77.5 (53.0; 100)	92.5 (75.5; 100)	90.0 (67.4; 100)	82.5 (61.8; 100)	*p *= 0.3^a^; *p *= 0.3^b^; *p *= 0.8^c^
Bodily pain	73.1 (53.9; 92.3)	75.9 (56.1; 95.7)	79.0 (60.7; 97.3)	85.0 (67.4; 100)	76.4 (59.3; 93.5)	73.7 (58.3; 89.1)	*p *= 0.2^a^; *p *= 0.1^b^; *p *= 0.1^c^
Physical functioning	76.0 (61.2; 90.8)	81.0 (65.7; 96.3)	79.5 (67.3; 91.7)	84.5 (71.2; 97.8)	80.1 (66.8; 93.3)	82.7 (72.4; 93.0)	*p *= 0.1^a^; *p *= **0.02**^**b**^; *p *= 0.1^c^
Mental health	80.8 (70.9; 90.7)	81.6 (69.5; 93.7)	81.6 (71.1; 92.1)	77.8 (67.7; 87.9)	84.8 (76.6; 93.0)	81.2 (70.2; 92.2)	*p *= 0.7^a^; *p *= 0.3^b^; *p *= 0.2^c^
Social functioning	93.8 (79.6; 100)	81.3 (64.8; 97.7)	93.8 (85.1; 100)	90.0 (78.2; 100)	95.0 (86.4; 100)	90.0 (76.8; 100)	*p *= 0.9^a^; *p *= 0.1^b^; *p *= 0.8^c^
Vitality	64.0 (53.0; 75.0)	68.5 (58.4; 78.6)	66.5 (56.5; 76.5)	60.0 (45.3; 74.7)	66.5 (52.6; 80.4)	66.5 (50.1; 82.9)	*p *= 0.8^a^; *p *= 0.7^b^; *p *= 0.9^c^

^a^Comparing baseline values between synbiotics and placebo group

^b^Comparing changes from baseline after 3 months of intervention between synbiotics and placebo group

^c^Comparing changes from baseline after 6 months of intervention between synbiotics and placebo group

Synbiotic effects were all transient and not detectable after 6 months without intervention. More information is given in Online Table 4.

### Dietary habits

Average daily protein, fat and digestible carbohydrates as well as fibre and total energy intake were estimated from a self-reported food frequency questionnaire. There were no significant changes between time points or differences between groups. Food intake did not correlate with glucose metabolism, or changes thereof. Details are given in Online Table 5.

## Discussion

In this pilot study, we show that the supplementation with a multispecies synbiotic in treatment-experienced diabesity patients had no effect on glucose metabolism, but could improve other aspects of diabesity. The presence of probiotic strains in the faecal microbiome seems to play a role in the facilitation and extent of beneficial effects.

The previously published benefits of probiotics on glucose metabolism could not be reproduced in this study. The possible reasons are manifold. The foremost difference between trials is the variation in the probiotic formulations, concentration and duration of intervention. Based on these heterogeneous trial parameters, a conclusive decision about the effects of probiotics as a whole cannot be made. However, even compared to trials of the same product the effects on metabolism in treatment-experienced obese diabetics were smaller than those in pre-diabetic and treatment-naïve diabetics [36, 37]. This raises the question of specific windows of opportunities for synbiotic interventions during the natural history of diabesity. Moreover, although the dropout rate was comparable between our study and the study of Sabico et al. [37], our pilot trial has considerably less statistical power which might obscure additional benefits for treatment-experienced patients.

The reduction of LPA in the synbiotics group amongst other unchanged lipid parameter reflects the conflicted literature on pro- and prebiotic effects on lipid profiles and our study could not reproduce the positive effects of microbiome modulation on lipoprotein-cholesterol shown in other reports [24, 26, 32]. Patients allocated to the synbiotics group experienced a temporary improvement in hip circumference. Synergistic effects of pro- and prebiotics on anthropometrics have been published before for overweight and obese adults, especially for the improvement of lean body mass and hip circumference [52]. The product we tested in this study was also associated with a reduction of waist-to-hip ratio or waist circumference and fat mass in two recent studies [36, 53]. Overall, however, the effects of probiotic interventions on anthropometric measurements are controversially discussed. While meta-analyses attest probiotics a modest benefit on body weight, other studies have observed an increase in liver fat content or body weight after probiotic intervention [54–57]. This suggests that the effects are product specific and should be tested separately for each formulation.

The study product was chosen for its favourable in vitro characteristics to strengthen gut barrier function [58]. Patients showed a significant reduction in gut permeability after 3 months of intervention as assessed by serum zonulin levels. Zonulin is an endogenous tight junction regulator and a common biomarker for gut permeability [59, 60]. It also correlates well with c-peptide, levels of LPS and bacterial DNA in serum in our patient collective. The improvement of gut permeability during probiotic supplementation is in accordance with previous studies: The use of the same multispecies probiotic as in the presented study in treatment-naïve T2D patients also led to a reduction in LPS levels after 6 months of intervention [37]. Additionally, a comparable multispecies product to the one used in this study has been reported to normalize zonulin levels in healthy trained men [61].

In a post hoc analysis of patients with detectable amounts of *L. brevis* in the gut microbiome after synbiotic intervention with the main objective to generate future research hypothesis, an improvement in glycaemic control was observed. *L. brevis* showed excellent ability to restore transepithelial electrical resistance after cytokine-induced barrier disruption in vitro and can produce anti-inflammatory cytokine IL-10 and alkaline phosphatase which can reduce endotoxin load in the intestine [58]. Although it is still unclear whether a measurable modulation of the microbiome is necessary for pro- or synbiotics to exert beneficial effects on clinical parameters, our data suggest that patients achieve better results when the intervention was associated with alterations of the microbiome composition. Also, a recently published study showed a strong association between microbiome modulation and improvement of glycaemic control following prebiotic intervention [28]. Anyway, it remains to be determined whether the presence of *L. brevis* in the presented study is a marker of true response or a marker of adherence. Poor adherence is a common problem in the treatment of T2D and obesity and might have declined after the first few months in our study [62]. This is corroborated by the fact that patients who showed an increase in *L. brevis* achieved better results in terms of HBA1c, endotoxemia and quality of life as well as that the most pronounced changes happened after 3 months of intervention and could not be sustained over the entire study period. A full-scale trial with appropriate power is warranted to validate these findings.

In addition, *L. brevis*^+^ patients in our study showed a reduction of serum LPS levels after 6 months of intervention, indicating decreased translocation of bacterial products. Low-grade “metabolic” endotoxemia is a key event in the development of metabolic syndrome and T2D, as was shown by Cani et al. [63] and has also consistently been demonstrated in diabetic subjects [63, 64]. Increased endotoxin levels may lead to innate immune dysfunction by overstimulation of neutrophil granulocytes [65]. In T2D and obesity, neutrophils react with less production of reactive oxygen species upon PMA stimulation compared to metabolically healthy obese individuals [66]. Furthermore, a tolerance to LPS has been described in animal models of diabetes and obesity [67]. This innate immune dysfunction contributes to the high risk of infections in T2D and obesity [68]. In our study, the synbiotics intervention increased production of reactive oxygen species by neutrophils. We have observed similar changes in neutrophil functionality in a previous study in patients with liver cirrhosis undergoing a 6-month intervention with the same product [47].

Quality of life is a clinically relevant outcome parameter and reflects the impact of health care interventions on the patients’ perspective and well-being [69, 70]. Probiotic use has been shown to increase quality of life in irritable bowel syndrome and patients with seasonal allergies [71–73]. Furthermore, synbiotic treatment could improve gastrointestinal quality of life in patients post-elective colorectal cancer resection [74]. In this study, patients showed a significant increase in quality of life, more specifically in ‘physical functioning’ of the SF-36 questionnaire, after 3 months of intervention compared to the placebo group. *L. brevis*^+^ patients maintained the improvement over 6 months of intervention. ‘Physical functioning’ reflects the ability to perform everyday activities, from carrying heavy objects and doing sports, over climbing sets of stairs and walking, to dressing and bathing [50]. An improvement in this area could potentially contribute to patients’ independence and promote physical activity, which has been linked to better glycaemic control before [75].

Nutrition plays an important role in diabesity and the composition of the microbiome. Pre- and synbiotic products have been shown to improve postprandial GLP-1 secretion and meal handling in diabetic patients and related SCFA production was linked to appetite regulation. Patients’ self-reported dietary habits did not change during the course of the study and did not correlate with the observed effects of the synbiotic.

The presented study has limitations. The dropout rate in this trial was 35% and, therefore, higher than anticipated. The main reason was withdrawal of consent which entailed a lack of motivation or energy to adhere to the extensive study protocol and long follow-up period. Only two patients (one in each group) cited side effects from the study preparation as the reason for dropout. This should be considered when interpreting the results of the study. Another limitation might be the uneven distribution of women in the study groups. This skewed some baseline parameters, such as height, weight, hip circumference, HDL-C and ApoA1, and unintentionally limits our conclusions mainly to male patients. The study was designed as a pilot study since the magnitude of changes in the microbial composition by probiotics in diabesity patients is largely unknown. Based on the relatively small effect sizes and conflicting results in the literature, large multicentre studies are warranted to reach definitive results.

In conclusion, synbiotics could not improve glucose metabolism in treatment-experienced diabesity patients in a 6 month intervention. The synbiotic could, however, improve secondary endpoints, including gut permeability and quality of life, which would make synbiotic supplementation a valuable add-on to the treatment of diabesity.

## Electronic supplementary material

Below is the link to the electronic supplementary material.

Supplementary material 1 (DOCX 450 kb)
