# Supplementary material for: Effects of a multispecies synbiotic on glucose metabolism, lipid marker, gut microbiome composition, gut permeability, and quality of life in diabesity: a randomized, double-blind, placebo-controlled pilot study
Source: Eur J Nutr. 2019 Nov 15;59(7):2969–83. doi: 10.1007/s00394-019-02135-w (PMC7501130; doi:10.1007/s00394-019-02135-w)
Supplement: Supplementary file 1 — Supplementary material 1 (DOCX 450 kb) [file 394_2019_2135_MOESM1_ESM.docx]

Online Resource European Journal of Nutrition

**Effects of a multispecies synbiotic on glucose metabolism, lipid marker, gut microbiome composition, gut permeability, and quality of life in Diabesity: A randomized, double-blind, placebo-controlled pilot study**

Angela Horvath,^1,2^ Bettina Leber,^3^ Nicole Feldbacher,^1,2^ Norbert Tripolt,^4^ Florian Rainer,^1^ Andreas Blesl,^1^ Markus Trieb,^5^ Gunther Marsche,^5^ Harald Sourij,^2,4^ Vanessa Stadlbauer^1^

^1^Division of Gastroenterology and Hepatology, Medical University of Graz, Graz, Austria

^2^Center for Biomarker Research in Medicine (CBmed), Graz, Austria

^3^Division of Transplantation Surgery, Medical University of Graz, Graz, Austria

^4^Division of Endocrinology and Diabetology, Medical University of Graz, Graz, Austria

^5^Division of Pharmacology, Otto Loewi Research Center, Medical University of Graz, Graz, Austria

**Sample collection, processing and storage**

Peripheral venous blood was aseptically collected at baseline and after three, six and twelve months into pyrogen-free tubes (VACUETTE®, Greiner Bio-One, Kremsmuenster, Austria) and kept at 4°C. For harvesting plasma blood was centrifuged at 2000xg for 10 min, for serum it was kept at room temperature for 30 minutes, and then centrifuged. After centrifugation, plasma or serum was aliquoted into non-pyrogenic cryotubes (Eppendorf, Hamburg, Germany) and stored at -80°C until further analysis.

Stool samples were collected into sterile tubes with a built-in sampling spatula by the patients at the day or the evening before the study visit and kept at 4°C. Upon the patient’s arrival at the hospital, samples were frozen immediately at 80°C until further analysis.

**Anthropometric measurements**

Weight was assessed on a common household scale without shoes or overcoat. Height was measured with a measuring tape fixed to the wall. BMI was calculated as weight in kg divided by the height in m squared. Waist circumference was measured with a flexible measuring band in standing position at the end of gentle exhalation. Hip circumference was also assessed in standing position over the great trochanters.

**Routine biochemistry measurements**

HbA1c was assessed by High Performance Liquid Chromtography (HPLC) on a Adams HA-8180V Analyzer (A. Menarini Diagnostics, Florence, Italy/Arkray, Kyoto, Japan). Plasma glucose is measured photometrically with an enzymatic reference method using hexokinase (Roche Diagnostics, Basel, Switzerland). Insulin and c-peptide were measured by chemiluminescence on an ADVIA Centaur system (Siemens Healthcare Diagnostics, Eschborn, Germany). HDL-C, LDL-C and VLDL-C was assessed via lipoprotein electrophoresis. Cholesterol and triglycerol were measured with an enzymatic colorimetric detection kit (Roche Diagnostics, Basel, Switzerland). Lipoprotein A, apolipoprotein A1 and apolipoprotein B were assessed using immunoturbidimetry (Roche Diagnostics, Basel, Switzerland).

**Online Table 1** Documented serious adverse events (SAE)

| Serious adverse event | Study phase | Causality | Group | Comments |
| --- | --- | --- | --- | --- |
| Biliary colic | active | unlikely | synbiotic | led to patients drop-out because of subjective overburdening |
| Decompensated heart failure | active | unlikely | placebo | led to patients drop-out because of subjective overburdening |
| Invasive gastroenteritis | active | unlikely | synbiotic | led to patients drop-out because of subjective overburdening |
| Hospitalization for pain management and impaired renal function | follow-up | unlikely | placebo |  |
| Cerebral tumor | follow-up | unlikely | placebo |  |
| Helicobacter pylori associated gastritis | follow-up | unlikely | placebo |  |
| Hospitalization for management of arterial hypertension | follow-up | unlikely | synbiotic |  |

**Online Table 2** Changes in glucose metabolism and lipid profile during intervention – additional parameters. Values are given as means (95% confidence interval)

|  | Synbiotics | | | Placebo | | |  |
| --- | --- | --- | --- | --- | --- | --- | --- |
|  | Baseline | 3 months | 6 months | Baseline | 3 months | 6 months | p-values |
| Early insulin response | 14.1 (1.8; 26.5) | 7.8 (-13.3; 28.9) | 4.7 (-7.6; 16.9) | 12.7 (2.6; 22.7) | 13.1 (3.0; 23.4) | 9.9 (-5.5; 25.4) | ^a^p=0.9; ^b^p=0.5; ^c^p=0.7; |
| Insulinogenic index | 96.3 (-12.9; 205.6) | 103.3 (-49.0; 255.5) | 37.8 (-38.4; 114.1) | 58.7 (15.5; 101.9) | -23.4 (-164.6, 117.7) | 65.7 (-48.4; 179.8) | ^a^p=0.9; ^b^p=0.8; ^c^p=0.4; |
| Matsuda index | 5.4 (1.1; 9.7) | 3.0 (1.6; 4.4) | 4.2 (0.0; 8.8) | 3.0 (1.8; 4.3) | 18.7 (0.0; 52.8) | 2.8 (1.7; 3.8) | ^a^p=0.9; ^b^p=0.9; ^c^p=0.6; |
| Quantitative insulin sensitivity check index | 0.32 (0.27; 0.37) | 0.28 (0.26; 0.30) | 0.28 (0.25; 0.30) | 0.29 (0.28; 0.31) | 0.28 (0.27; 0.30) | 0.29 (0.27; 0.31) | ^a^p=0.8; ^b^p=0.6; ^c^p=0.5; |
| First phase of insulin secretion | 864 (46; 1682) | 1343 (-483; 3169) | 2161 (187; 4135) | 811 (376; 1246) | 845 (390; 1300) | 614 (-100; 1328) | ^a^p=0.8; ^b^p=0.9; ^c^p=0.2; |
| Second phase of insulin secretion | 267 (74; 460) | 390 (-39; 818) | 583 (114; 1052) | 256 (155; 356) | 268 (162; 374) | 214 (55; 374) | ^a^p=0.9; ^b^p=0.9; ^c^p=0.2; |
| Cholesterol (mg/dl) | 166 (140; 192) | 161 (137; 185) | 171 (137; 205) | 181 (154; 208) | 191 (169; 212) | 180 (160; 201) | ^a^p=0.3;  ^b^p=0.4; ^c^p=0.9; |
| Cholesterol efflux (%) | 8.32 (7.44; 9.21) | 8.43 (7.30; 9.57) | 8.80 (7.74; 9.86) | 9.42 (8.65; 10.19) | 9.58 (8.66; 10.52) | 9.29 (8.46; 10.12) | ^a^p=0.1; ^b^p=0.7; ^c^p=0.2; |
| Triglycerides (mg/dl) | 182 (104; 260) | 171 (124; 219) | 175 (114; 235) | 158 (95; 221) | 354 (0; 800) | 143 (104; 182) | ^a^p=0.6 ^b^p=0.7; ^c^p=0.9; |
| LPA (mg/dl) | 18.0 (5.9; 30.0) | 18.9 (5.4; 32.4) | 15.6 (7.2; 24.1) | 17.4 (6.6; 28.3) | 20.3 (7.5; 33.2) | 20.9 (7.2; 34.6) | ^a^p=0.8 ^b^p=0.2; **^c^p=0.02;** |
| HDL-C (mg/dl) | 35 (29; 41) | 40 (30; 50) | 38 (31; 45) | 47 (38; 57) | 47 (32; 62) | 45 (35; 54) | **^a^p=0.02** ^b^p=0.2; ^c^p=0.2; |
| LDL-C (mg/dl) | 95 (77; 113) | 90 (71; 109) | 101 (79; 123) | 109 (86; 132) | 104 (87; 122) | 103 (82; 123) | ^a^p=0.9 ^b^p=0.3; ^c^p=0.9; |
| HDL-C/LDL-C | 0.42 (0.24; 0.60) | 0.49 (0.31; 0.66) | 0.40 (0.31; 0.48) | 0.51 (0.32; 0.71) | 0.51 (0.31; 0.71) | 0.50 (0.32; 0.68) | ^a^p=0.08 ^b^p=0.3; ^c^p=0.5; |
| VLDL-C (mg/dl) | 35 (21; 50) | 29 (20; 38) | 31 (19; 43) | 26 (18; 35) | 44 (10; 78) | 30 (20; 40) | ^a^p=0.4; ^b^p=0.6; ^c^p=0.3; |
| ApoA1 (mg/dl) | 146 (134; 160) | 155 (139; 172) | 154 (138; 171) | 160 (147; 174) | 169 (154; 184) | 167 (151; 184) | ^a^p=0.06 ^b^p=0.9; ^c^p=0.9; |
| ApoB (mg/dl) | 84 (69; 99) | 84 (70; 97) | 90 (72; 108) | 91 (73; 108) | 99 (77; 121) | 94 (81; 107) | ^a^p=0.7; ^b^p=0.3; ^c^p=0.9; |

MTT: meal tolerance test; HDL-C: high-density lipoprotein cholesterol; LDL-C: low-density lipoprotein cholesterol; VLDL-C: very low-density lipoprotein cholesterol; ApoA1: apolipoprotein A1; ApoB: apolipoprotein B; ^a^comparing baseline values between synbiotics and placebo group; ^b^comparing changes from baseline after three months of intervention between synbiotics and placebo group; ^c^comparing changes from baseline after six months of intervention between synbiotics and placebo group

**Online Table 3** Detailed information about glucose lowering medication according to allocation

|  | Synbiotics | Placebo | p-value |
| --- | --- | --- | --- |
| Insulin |  |  |  |
| +Metformin | 0 (0%) | 2 (14%) |  |
| +SGLT-2 inhibitors | 0 (0%) | 1 (7%) |  |
| no combination | 1 (8%) | 1 (7%) |  |
| -in total | 1 (8%) | 4 (29%) | 0.19 |
| DPP4 inhibitors |  |  |  |
| +Metformin | 1 (8%) | 2 (14%) |  |
| +Pioglitazone | 0 (0%) | 1 (7%) |  |
| +Sulfonylurea | 1 (8%) | 0 (0%) |  |
| +Metformin, Pioglitazone | 0 (0%) | 1 (7%) |  |
| +Metformin, Sulfonylurea | 1 (8%) | 0 (0%) |  |
| +Metformin, Pioglitazone, Sulfonylurea | 2 (17%) | 0 (0%) |  |
| -in total | 5 (42%) | 4 (29%) | 0.48 |
| Insulin and DPP4 inhibitors |  |  |  |
| +Metformin | 2 (17%) | 4 (29%) |  |
| +Metformin, SGLT-2 inhibitors | 2 (17%) | 0 (0%) |  |
| -in total | 4 (33%) | 4 (29%) | 0.79 |
| Other therapy regimes |  |  |  |
| +Metformin, Sulfonylurea | 0 (0%) | 1 (7%) |  |
| +Metformin, SGLT-2 inhibitors | 1 (8%) | 1 (7%) |  |
| +Metformin | 1 (8%) | 0 (0%) |  |
| -in total | 2 (17%) | 2 (14%) | 0.87 |

SGLT-2: sodium-glucose co-transporter 2; DPP4: Dipeptidyl peptidase-4

**Online Table 4** Modulated parameters after six months of follow-up. Values are given as means (95% confidence interval)

|  | Synbiotics | | Placebo | |  |
| --- | --- | --- | --- | --- | --- |
|  | Baseline | Follow-up | Baseline | Follow-up | p-values^a^ |
| Hip circumference (cm) | 117 (112; 123) | 115 (110; 121) | 111 (107; 115) | 112 (105; 118) | p=0.4 |
| LPA (mg/dl) | 18.0 (5.9; 30.0) | 17.8 (0; 37.0) | 17.4 (6.6; 28.3) | 21.4 (6.9; 35.8) | p=0.1 |
| Zonulin (ng/ml) | 2.45 (2.04; 2.85) | 2.63 (2.0; 3.27) | 2.01 (1.62; 2.41) | 2.45 (2.13; 2.77) | p=0.07 |
| Physical functioning | 76.0 (61.2; 90.8) | 77.5 (62.8; 92.2) | 84.5 (71.2; 97.8) | 85.8 (74.3; 97.4) | p=0.3 |

LPA: lipoprotein (a); ^a^comparing changes from baseline between synbiotics and placebo group

**Online Table 5** Average self-reported nutrient intake. Values are given as means (95% confidence interval)

|  | Synbiotics | | | Placebo | | |  |
| --- | --- | --- | --- | --- | --- | --- | --- |
|  | Baseline | 3 months | 6 months | Baseline | 3 months | 6 months | p-values |
| Energy intake (kcal/day) | 1586 (1202; 1970) | 1539 (1205; 1873) | 1352 (1012; 1693) | 1590 (1107; 2073) | 1690 (1152; 2227) | 1408 (1063; 1753) | ^a^p=0.9; ^b^p=0.8; ^c^p=0.5; |
| Protein intake (g/day) | 70 (53; 87) | 67 (53; 81) | 59 (45; 74) | 72 (51; 94) | 77 (52; 101) | 68 (47; 89) | ^a^p=0.9;  ^b^p=0.7; ^c^p=0.4; |
| Fat intake (g/day) | 56 (41; 71) | 54 (42; 67) | 54 (36; 71) | 61 (39; 82) | 63 (43; 82) | 55 (34; 76) | ^a^p=0.7; ^b^p=0.8; ^c^p=0.9; |
| Digestable carbohydrate intake (g/day) | 191 (132; 250) | 189 (137; 241) | 151 (109; 193) | 180 (112; 248) | 198 (120; 276) | 153 (114; 192) | ^a^p=0.6; ^b^p=0.9; ^c^p=0.4; |
| Fibre intake (g/day) | 28 (20; 36) | 26 (18; 33) | 21 (16; 27) | 27 (17; 36) | 29 (16; 42) | 24 (17; 31) | ^a^p=0.6; ^b^p=0.8; ^c^p=0.2; |

^a^comparing baseline values between synbiotics and placebo group; ^b^comparing changes from baseline after three months of intervention between synbiotics and placebo group; ^c^comparing changes from baseline after six months of intervention between synbiotics and placebo group

Online Figures


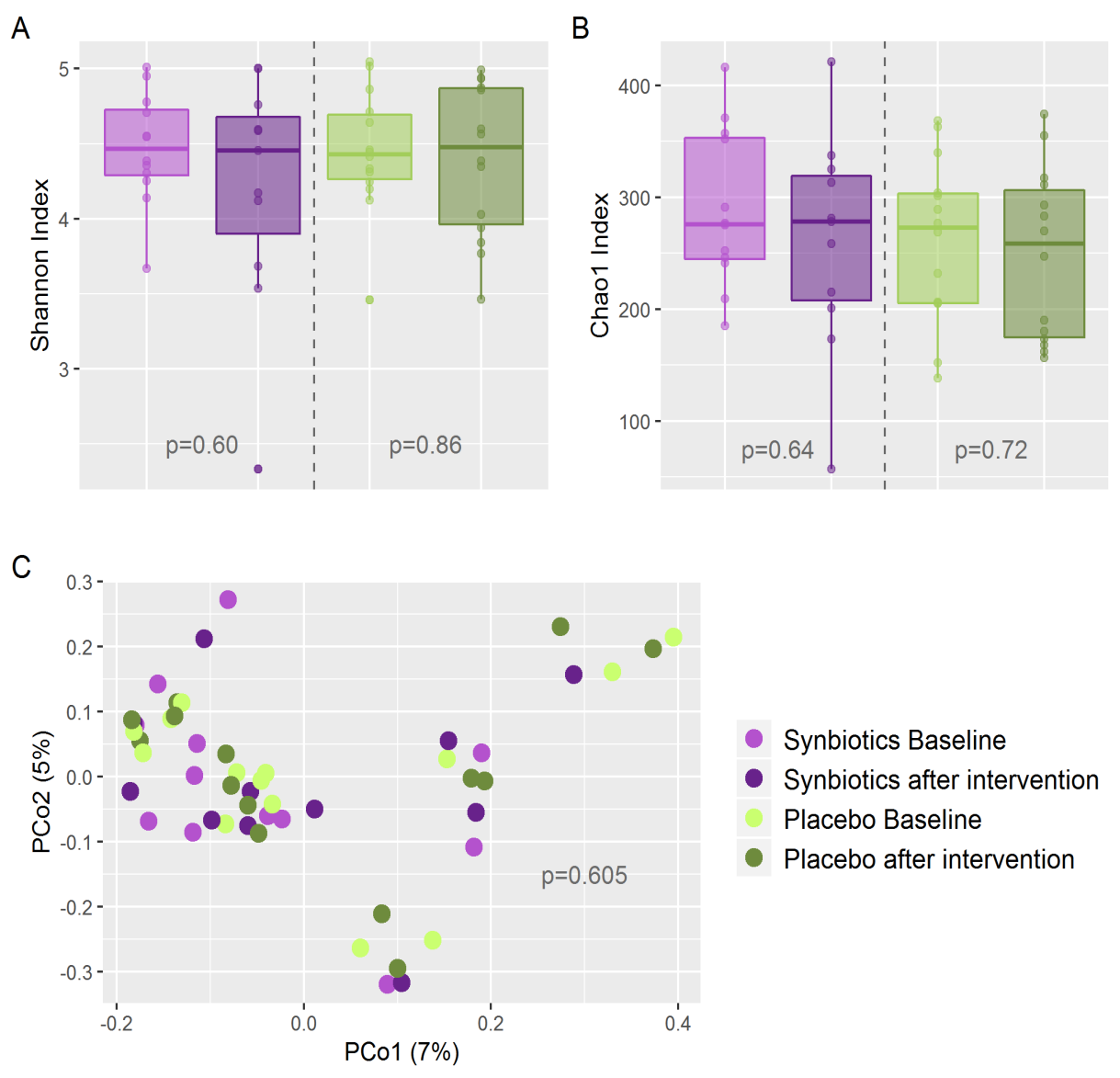


Online Figure 1: Alpha and beta diversity measures of the stool microbiome before and after intervention for both synbiotic and placebo group. A: Alpha diversity assessed by Shannon index; B: Richness of the microbiome assessed by Chao1 index; C: Beta diversity based on Bray-Curtis dissimilarity presented in a principal coordinate analysis plot. Legend is appropriate for all panels. Graphs were produced using R3.6 and the packages “ggplot2” and “ggpubr” [1-3].


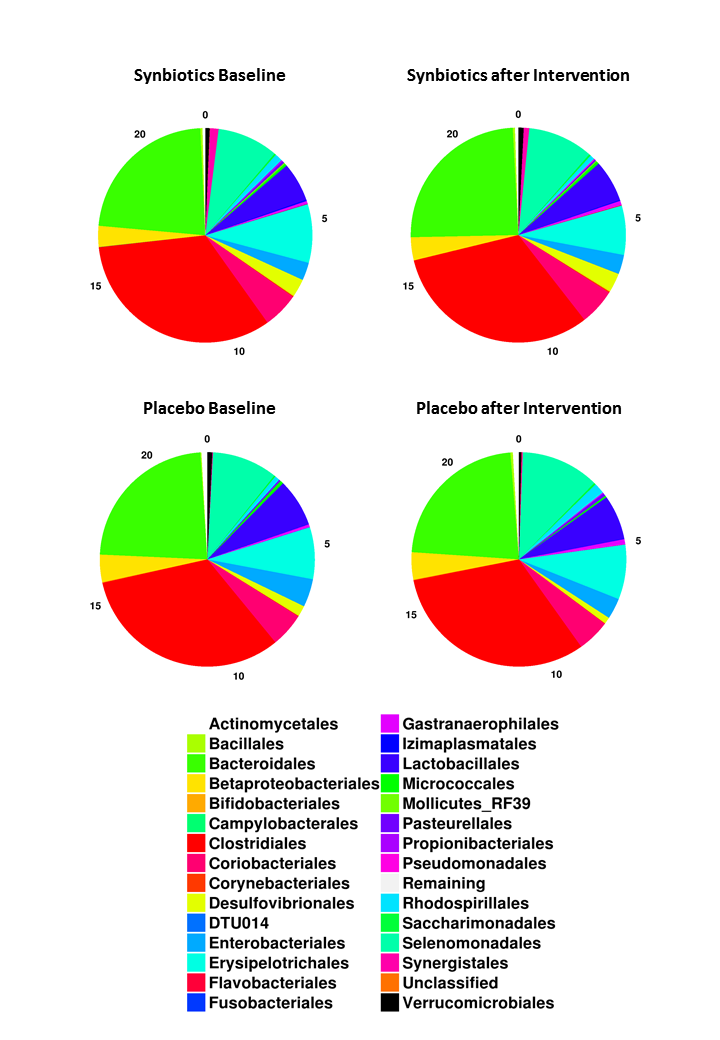


Online Figure 2: Microbiome composition data before and after the intervention for both synbiotic and placebo group. Taxonomy is given on order level. Graphs were produced by the Calypso 8.84 [4].

References:

1. 'R_Core_Team' (2017) R: A language and environment for statistical computing. R Foundation for Statistical Computing, Vienna, Austria. In:

2. Wickham H (2009) ggplot2: Elegant Graphics for Data Analysis. Springer-Verlag New York

3. Kassambara A (2018) ggpubr: 'ggplot2' Based Publication Ready Plots. In:

4. Zakrzewski M, Proietti C, Ellis JJ, Hasan S, Brion MJ, Berger B, Krause L (2017) Calypso: a user-friendly web-server for mining and visualizing microbiome-environment interactions. Bioinformatics (Oxford, England) 33:782-783. doi 10.1093/bioinformatics/btw725.
